# COVID-19 Assessment and Testing in Rural Communities During the Pandemic: Cross-sectional Analysis

**DOI:** 10.2196/30063

**Published:** 2022-02-08

**Authors:** Jonathan Fitzsimon, Oliver Gervais, Chelsea Lanos

**Affiliations:** 1 Department of Family Medicine University of Ottawa Ottawa, ON Canada; 2 Arnprior Regional Health Arnprior, ON Canada; 3 Department of Family Medicine Discipline of Emergency Medicine Memorial University St. John's, NL Canada; 4 County of Renfrew Paramedic Service Renfrew, ON Canada

**Keywords:** healthcare, virtual care, access, COVID-19, pandemic, assessment, testing, community paramed, digital health, online health, physician, virtual health

## Abstract

**Background:**

The COVID-19 pandemic exacerbated the need for urgent improvements in access to health care for rural, remote, and underserviced communities. The Renfrew County Virtual Triage and Assessment Centre (VTAC) was designed to provide access to COVID-19 testing and assessment with a family physician. The goal was to protect emergency departments and 911 paramedics while ensuring that nobody was left at home, suffering in silence. Residents were encouraged to call their own family physician for any urgent health needs. If they did not have a family physician or could not access their usual primary care provider, then they could call VTAC. This study reports on the output of a service model offering access to assessment and COVID-19 testing through a blend of virtual and in-person care options by a multidisciplinary team.

**Objective:**

The purpose of this study was to assess the ability of VTAC to provide access to COVID-19 assessment and testing across rural, remote, and underserviced communities.

**Methods:**

We conducted a cross-sectional analysis of the data derived from the cases handled by VTAC between March 27, 2020 (launch day), and September 30, 2020.

**Results:**

Residents from all 19 census subdivisions and municipalities of Renfrew County accessed VTAC. A total of 10,086 family physician assessments were completed (average 64 per day). Of these, 8535 (84.6%) assessments were to unique patient users. Thirty physicians provided care. Using digital equipment setup in the patients’ home, 31 patients were monitored remotely. VTAC community paramedics completed 14,378 COVID-19 tests and 3875 home visits.

**Conclusions:**

Renfrew County’s experience suggests that there is tremendous synergy between family physicians and community paramedics in providing access to COVID-19 assessment and COVID-19 testing. The blended model of virtual and in-person care is well suited to provide improved access to other aspects of health care post pandemic, particularly for patients without a family physician.

## Introduction

On March 11, 2020, the World Health Organization announced that the novel coronavirus outbreak first identified in China could be characterized as a pandemic [1]. Since then, the World Health Organization released a response plan to help countries prevent and delay outbreaks and improve patient care [2]. In response to this, the Ontario provincial government asked local health authorities to establish COVID-19 assessment centers [3].

In many urban settings, large buildings were repurposed, allowing health care workers to assess patients and perform COVID-19 testing. While functional and realistic for larger, densely populated areas, this was impractical for rural and remote areas. Patients suspected of having COVID-19 in these regions often relied on emergency departments (EDs) to provide basic care, threatening to increase risk of community spread, reduce capacity for responding to genuine emergencies (including severe symptoms due to COVID-19 infection), and dissuade patients from seeking treatment for other conditions for fear of infection [4].

We created the Renfrew County Virtual Triage and Assessment Centre (VTAC) to improve access to COVID-19 assessment and testing in rural, remote, and underserviced communities. Through a local advertising campaign, using social media, radio interviews, and roadside notice boards, the residents were encouraged to contact their own family physician for urgent health needs. Those without a family physician or unable to reach their usual primary care provider could call VTAC. Patients call a toll-free telephone number and speak to a trained medical receptionist, with experience working in a physician’s office, who could provide advice and schedule appointments with a physician when required. Family physicians provide virtual care by telephone and video encounters. Community paramedics offer COVID-19 testing at scheduled drive-through sites, ad hoc pop-up sites, and in-home settings for vulnerable housebound patients. Referral to existing community health care services enables patients to access a wide array of community support without resorting to the ED.

The primary purpose of this paper was to describe and quantify the output of the Renfrew County VTAC in the first 6 months of operation, from March 2020 to September 2020. We anticipate that our findings could help improve VTAC service delivery in Renfrew County and guide the implementation of similar systems in other communities. Secondarily, we gathered data that highlight the impact an ongoing system such as VTAC might have on increasing access to care for rural and underserved populations.

## Methods

### Study Design

We conducted a cross-sectional analysis of the data derived from the cases handled by VTAC between March 27, 2020 (launch day), and September 30, 2020.

### Setting

Renfrew County is the largest geographic county in Ontario, encompassing almost 7500 km^2^, with a population of approximately 107,000. Five larger towns in the county have community hospitals (including ED). A chronic shortage of family physicians has resulted in gross inequalities in access to primary care across the county. There are no walk-in clinics, and there is an enormous overreliance on ED as a means of accessing any form of health care.

### VTAC Development

Close integration of primary care and paramedic services in the region allowed for a model where paramedics would undertake all COVID-19 testing. Initially carried out entirely in patients’ homes, this testing model gradually expanded to include pop-up sites for small groups and eventually various prescheduled drive-through sites across the county. Vulnerable housebound patients and those unable to attend a drive-through site were still able to be tested at home by community paramedics. The key message given to the public was, “If you have a health concern, call your family physician first. If you do not have a family physician or cannot access your family physician, then call VTAC.”

### VTAC Model

**Figure 1:**
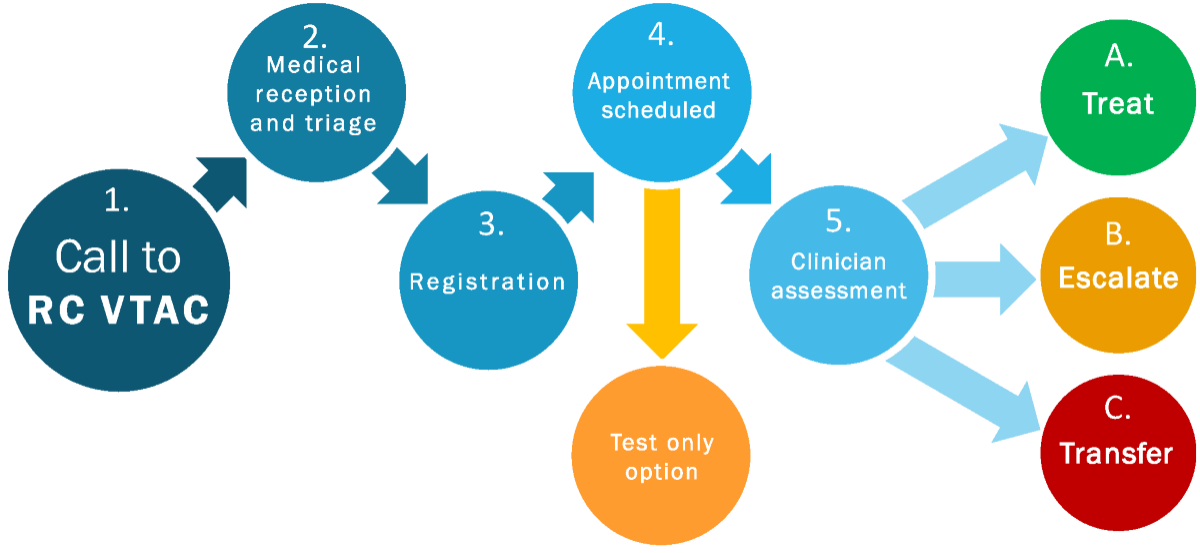
VTAC patient flow. RC VTAC: Renfrew County Virtual Triage and Assessment Centre

Patients accessed VTAC by calling a toll-free phone number (Figure 1). A medical receptionist recorded the patient’s demographic and other relevant information, including whether the patient had an existing family physician. For first time callers to VTAC, the receptionist created a basic medical chart in the electronic medical records (EMRs). Previous medical information could be retrieved electronically through Ontario’s existing eHealth systems, Clinical Viewer and the Ontario Laboratories Information system.

Digital home monitoring equipment could be left in the patient’s home, with community paramedics completing in-person patient teaching on how to use the equipment. Paramedics and a VTAC physician could then monitor the patients remotely and be available for check-ins at short notice. Clear referral pathways were created so that VTAC physicians could refer patients to the existing community health care resources such as mental health support and community palliative care as well as specialist services via electronic or telephone referral processes. Once all of these options are exhausted, VTAC physicians can advise patients to go to the ED or call 911 (Figure 1).

Physicians are compensated using Ontario’s COVID-19 billing codes, created specifically for designated COVID-19 assessment centers [5]. The costs for paramedics, receptionists, and other administration are covered through Ontario’s assessment center funding and managed by one of the hospital chief executive officers.

Thirty physicians provided care through VTAC. Most were physicians with an existing practice based in Renfrew County. Others worked in nearby Ottawa but had experience of working in Renfrew County through previous residency rotations or locum positions. Time commitments ranged from a few hours each week to regular, multiple shifts each week.

### Data Collection

Despite multiple attempts over many years, it has not been possible to obtain accurate or complete data regarding the number of residents who do not have a dedicated primary care provider (PCP). Therefore, family physicians and managers from primary care and hospital settings gathered data on the number of family physicians currently practicing in Renfrew County and their roster size. Primary care groups reported the number of patients with a nurse practitioner as their PCP. Of the 77 family physicians currently practicing in Renfrew County, 70 (90%) returned roster sizes from their EMR (individually or through their group EMR administrator). Moreover, 4 (5%) family physicians estimated their roster size as they currently use paper charts, and 3 (3%) family physicians did not respond, in which case, local colleagues estimated their roster size.

We used routine utilization data for VTAC services in Renfrew County, which included the following elements: numbers of physician assessments; paramedic home visits; COVID-19 tests performed; physician assessments for patients without a family physician or alternate PCP; and gender, age, and postal code. Data were collected from the EMRs of VTAC patients by running a series of reports using built-in EMR capabilities, then transferred to Excel spreadsheets to identify repeat users, as well as the total number of events and unique patient users. For patients with an existing family physician, a copy of the VTAC encounter note was sent to their family physician to ensure integration with their regular EMR. We also cross-referenced postal codes with data from the 2016 Census [6] and IntelliHealth Ontario [7]. This allowed the evaluation of VTAC usage from each census subdivision within Renfrew County.

### Analysis

The analysis of data on the population of Renfrew County showed a small difference in the published figures from the 2016 census (102,394) [6] and IntelliHealth’s 2020 report (107,286) [7]. We used the IntelliHealth data as the more recent review for results and cost analysis. We further analyzed the VTAC data sets to establish a more detailed demographic breakdown of age and gender.

We evaluated cost by reviewing VTAC budget reports of administrative costs and nonphysician staff costs, as well as billings submitted by VTAC physicians to the Ministry of Health. At the start of the pandemic, there was a willingness and availability of clinical, administrative, and management staff from hospitals, primary care teams, and other health care services to provide support in-kind to the efforts to combat the pandemic and provide a COVID-19 assessment center for the community. In October 2020, a new funding model for assessment centers was approved by the Government of Ontario [8]. Our budget planning moving forward includes the costs for support and services that have been partially or fully offered in-kind during the initial pandemic period. Currently, there is a more stable prediction of future costs based on observation of trends, volumes, and usage of the service over 6 months of operation since inception.

### Ethics Approval

Based on criteria provide by the Winchester District Memorial Hospital Research Ethics Board, this project was undertaken as a quality improvement initiative; thus, review by a research ethics board was not needed.

## Results

**Figure 2:**
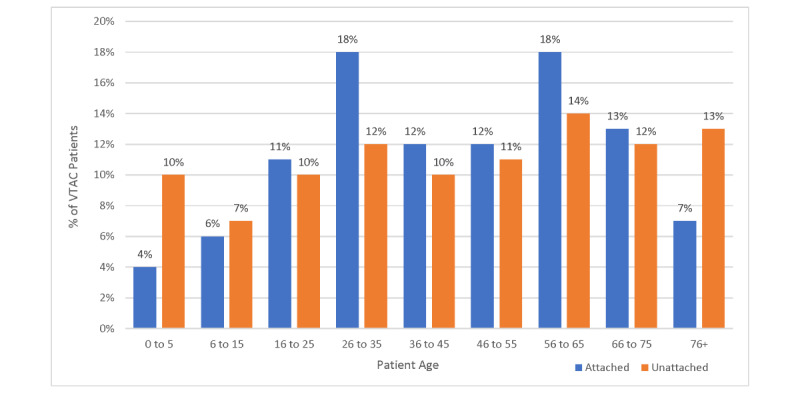
Distribution of ages of VTAC patients unattached (n=3641) and attached (n=4894) to a primary care provider. VTAC: Virtual Triage and Assessment Centre.

There were 82,450 patients registered with a practicing family physician and 2070 with a nurse practitioner, indicating that 22,766 residents (21.2% of Renfrew County residents) were not registered with a PCP. Patients with a family physician were slightly older on average than patients without a family physician (Figure 2), with 25% (n=1223) of attached patients being over the age of 65 versus 20% (n=728) of unattached patients. The proportion of male and female patients was similar between groups (62% [n=3034] of attached patients were female vs 58% [n=2111] of unattached patients).

### VTAC Usage

Residents from all census subdivisions and municipalities used VTAC. A total of 10,086 family physician assessments were completed, an average of 64 assessments per day. Of these, 8535 (84.6%) assessments were to unique patient users, of whom 3641 (42.7%) did not have a PCP (Figure 2).

Approximately one-third of the encounters were specifically COVID-19–related, and two-thirds were not directly COVID-19–related. This reduced unnecessary ED visits and the associated risks of infection and spread of COVID-19 within ED. Only 3% (302 of 10,086) of VTAC assessments resulted in a transfer to ED or 911.

Community paramedics supported by a VTAC physician remotely monitored 31 patients. A total of 1058 remote assessment days were completed, with many of these patients being monitored closely to prevent transfer to ED or admission to hospital. VTAC community paramedics completed 14,378 COVID-19 tests, including 3875 home visits.

### The Cost of VTAC

The total cost of establishing and operating VTAC up to September 30, 2020, was $2,695,725. The actual costs comprised community paramedics ($1,262,042), physician fees ($904,030), medical receptionists ($266,364), information technology and communications ($40,775), medical equipment ($31,257), and other administrative support ($11,256). In-kind support had an estimated value of $180,000, which included management and administrative support ($120,000); internet-based telephone system and tech support from a partnership with Ontario 211 community and social services ($28,000); and infrastructure support from Telus, such as the use of the Practice Solutions Suite EMR situated at the Arnprior and District Family Health Team and reduction or waiving of various usual fees ($32,000). A currency exchange rate of CAD $1=US $0.78 is applicable.

By using this EMR from the beginning, VTAC was able to establish several basic tracking forms to enable subsequent data collection. VTAC was used by 22,944 unique patients and was available to all residents. Overall, the total cost was equivalent to $25.13 for every resident of Renfrew County for the setup and first 6 months of operating costs.

## Discussion

### Principal Findings

The primary aim of this manuscript was to describe and quantify the output of the Renfrew County VTAC in the first 6 months of operation. During that period, VTAC was accessed by nearly 23,000 unique patients for COVID-19 testing, virtual family physician assessment, community paramedic in-home assessment, or remote assessment using in-home monitoring equipment. Residents in rural, remote, urban, and First Nation communities who have used VTAC reported a high level of patient acceptability and satisfaction with the service. In addition to providing access to COVID-19 testing and assessment, VTAC has provided a vital and potentially cost-effective alternative to the ED for residents who do not have a primary care provider or are unable to access their PCP.

VTAC has attracted significant amounts of media attention. Local political leaders have fully endorsed VTAC due to the contribution it has made to the population of Renfrew County during the pandemic and the overwhelming desire of the community for the service to continue [9]. Moreover, VTAC has been shown to be highly accepted by its users. In a recent study among 219 VTAC patients, 86% reported that their concern was dealt with at the first virtual encounter, 93% reported being happy or very happy with the service, and 98% would recommend VTAC to family and friends. In addition, 46% of the 219 VTAC users reported that without VTAC, they would have attended an ED [10]. Therefore, since its implementation, VTAC has proven to be an effective vehicle for delivering high-quality acute, episodic care in rural, remote, and underserviced communities.

Previous articles have described aspects of virtual care during the COVID-19 pandemic [11-17]. Some Canadian provinces have implemented virtual walk-in clinics, providing assessment by a physician through a mobile phone app [18]. One study described the expansion of a United States virtual telehealth program, which provides 24/7 access to a virtual acute care physician [16]. Another article described the triage and assessment of suspected COVID-19 patients originating from ED, hospital discharges, and PCPs. Patients determined to be safe for discharge were admitted to a “Virtual Ward” (ie, their home) for remote oximetry monitoring. Local paramedics could be called to arrange transport to hospital at any time, based on preestablished criteria suggestive of deterioration. The study concluded that this was a safe method for managing COVID-19 patients, and there were overall significant cost savings [19]. Although these virtual programs have some similarities to VTAC, VTAC is unique in its combination of many different new and innovative approaches to create a comprehensive virtual triage, assessment, and monitoring program for patients who do not have access to a PCP. These approaches include a tight partnership producing tremendous synergy between primary care and community paramedicine, the implementation of virtual care technologies (including remote monitoring), a close collaboration with hospitals and public health, and a partnership with a wide array of preexisting health care and community resources.

However, VTAC is not a replacement for comprehensive primary care. Complex health issues, such as chronic pain management, are poorly suited to virtual, episodic care [18]. Some complaints, including some musculoskeletal and neurological symptoms, are generally not amenable to virtual care [20]. Moreover, VTAC does not replace the continuity, depth, and breadth of care that is offered by a regular family physician and a strong, long-term doctor-patient relationship. However, our results showing that 21.2% of Renfrew County residents do not have a PCP are in keeping with the 2020 Canadian survey data, which found that 20% of Canadians have no family doctor [21]. The problem is exacerbated in Renfrew County by the absence of any walk-in clinics. The Canada-wide lack of access to PCPs strengthens the potential benefits that a program similar to VTAC could have in other communities. In addition to providing an alternative to the ED for those who do not have access to a PCP, virtual care has been shown to be cost-effective from both health and societal perspectives [11,22].

Notably, the VTAC model is well suited to assist with a COVID-19 community mass vaccination program. Community paramedics have implemented highly effective and efficient drive-through sites where they currently perform COVID-19 testing. It is within the scope of practice of the community paramedics to administer vaccinations. The physical sites are easily adaptable to include in-vehicle waiting areas (to monitor patients and be immediately available to deal with any postvaccination side effects). From the outset, the sites were “winterized” and can be capable of functioning in a range of weather conditions. These drive-through sites can accommodate large numbers of people in a timely manner and have the capacity to expand quickly to meet surges in demand.

The VTAC model could also provide a basis of longer-term health care support to the underserviced population by providing an alternative to the ED for patients who do not have a family physician but require regular, chronic disease management. Urgent care can also be provided to patients without a family physician or when a patient’s regular family physician is not available. One of the key benefits of having a family physician make these assessments is that they are ideally placed to identify high risk signs and symptoms that do warrant emergency care. This reassures patients that their need for emergency care is justified and warranted while helping to protect emergency services for genuine emergencies. Family physicians are also well versed in managing risk and being able to explain clear safety netting steps to patients so that they understand the circumstances where additional or repeat assessment would be required.

### Strengths and Limitations

Our study has several limitations. It was conducted in 1 region (Renfrew County), and its findings might not be generalizable to other settings. There are limitations which are inherent to virtual care, including challenges obtaining vital signs, limited ability to perform a physical exam and subsequent risk of misdiagnosis [15]. Despite these limitations, VTAC’s overwhelmingly positive patient feedback is comparable to the positive satisfaction rates noted in other telemedicine studies [11,12,23,24]. As COVID-19 risk stratification scores such as RECAP-V0 (Remote COVID-19 Assessment in Primary Care) and DDC19 (dynamic risk assessment decision support system for COVID-19) are further validated, we hope to incorporate these into our assessments, helping to more objectively determine which patients are at the highest risk of deterioration [25,26].

Detailed information regarding VTAC video appointments were not specifically collected. Therefore, we cannot comment on the proportion of video visits during the study period, or what patient issues or conditions led to video or telephone appointments. This was, in part, due to the overwhelming majority of VTAC encounters being sufficiently dealt with and completed by phone. However, VTAC physicians were able to use a secure video platform if required. Furthermore, although VTAC was accessible for both COVID-19 and non–COVID-19 related issues, no specific insights were available for a detailed analysis of the suitability of VTAC by health concern. Further research is underway to follow up with VTAC physicians and analyze these issues. Lastly, while we were able to estimate the total cost equivalent for VTAC for the setup and first 6 months of operating (cost for every resident of Renfrew County), a detailed evaluation of the economic impact on overall health care costs was beyond the scope of this paper. Future research aiming to evaluate both the clinical and economic impacts of VTAC in Renfrew County is warranted.

### Conclusion

The introduction of VTAC in Renfrew County has greatly enhanced access to COVID-19 assessment and testing in rural communities during the pandemic, especially for individuals unattached to a primary care provider. Virtual care has proven to be overwhelmingly acceptable to VTAC patients and has improved their experience of health care and health outcomes. Physicians have quickly adapted to different techniques and benefitted from the advantages of being able to provide care despite the restrictions of the pandemic. When compared to the costs of 911 transfer, ED attendance, and hospital admissions, VTAC may provide a highly cost-effective improvement to the overall health care system. The VTAC structure is also well suited to assist with a COVID-19 community mass vaccination program; once the COVID-19 pandemic eventually subsides, the need for COVID-19 assessment centers will diminish. Furthermore, the VTAC model could provide a basis of health care support to the underserviced population; it can also be a stepping-stone toward a situation whereby all Canadians have access to a family physician and comprehensive primary care. Future research should be aimed at assessing the clinical and economic impacts of the implementation and ongoing use of VTAC in Renfrew County.

## Abbreviations

DDC19: dynamic risk assessment decision support system for COVID-19
ED: emergency department
EMR: electronic medical record
PCP: primary care provider
RECAP: Remote COVID-19 Assessment in Primary Care
VTAC: Virtual Triage and Assessment Centre
